# First-line chemotherapy with liposomal doxorubicin plus cisplatin for patients with advanced malignant pleural mesothelioma: phase II trial

**DOI:** 10.1038/bjc.2012.44

**Published:** 2012-02-21

**Authors:** Ó Arrieta, L A Medina, E Estrada-Lobato, N Hernández-Pedro, G Villanueva-Rodríguez, L Martínez-Barrera, E O Macedo, V López-Rodríguez, D Motola-Kuba, J F Corona-Cruz

**Affiliations:** 1 Clinica de Tumores Torácicos, San Fernando #22, Colonia Sección XVI, Tlalpan, México City 14080, México; 2 Laboratorio de Oncología Experimental, Instituto Nacional de Cancerología (INCan), San Fernando #22, Colonia Sección XVI, Tlalpan, México City 14080, México; 3 Instituto de Física, Universidad Nacional Autónoma de México (UNAM), México City, México; 4 Unidad de Investigación Biomédica en Cáncer, INCan-UNAM, México City, México; 5 Departamento de Medicina Nuclear, INCan, México City, México; 6 Departamento de Oncología, Instituto Nacional de Enfermedades Respiratorias (INER), México City, México

**Keywords:** malignant pleural mesothelioma, liposomal doxorubicin, quallity life, cisplatin, Phase II, LD radiolabelling

## Abstract

**Background:**

Chemotherapy based on platinum is the standard treatment for unresectable malignant pleural mesothelioma (MPM). Liposomal doxorubicin (LD) consists of pegylated phospholipid vesicles that encapsulate doxorubicin-enhancing liposome deposition in the tumour. We evaluated the toxicity profile and anti-tumour activity of cisplatin plus LD in untreated patients with MPM, as well as ^99m^Tc-LD distribution in MPM lesions after chemotherapy administration.

**Methods:**

A total of 38 patients with non-resectable MPM received LD 40 mg m^−2^ and cisplatin 60 mg m^−2^ every 21 days. Gamma camera images of ^99m^Tc-LD were acquired to evaluate LD accumulation in measurable tumour tissue. The study was registered in Clinical Trials (NCT00886028).

**Results:**

In all, 72% of patients were stage III and 28% were stage IV. Eighty four percent and 16% have high and low risk acording EORTC respectively. The median time to progression was 4.6 months (95% confidence interval (95% CI: 3.4–5.9 months), and median overall survival (OS) was 19.6 months (15.2–37.2 months). Patients that responded to chemotherapy treatment had better survival than patients who did not. Functional physical scales, dysnea, cough, and chest/arm pain demonstrated improvement. The accumulation ratio of LD in tumour and soft tissues *vs* liver was 0.78±0.16 and 0.29±0.09, respectively. After 1 h of administration, LD uptake in tumour tissue was higher than in soft tissue (*P*< 0.001).

**Conclusion:**

The combination of LD and cisplatin results in an active therapeutic regimen for unresectable MPM, with an acceptable toxicity profile and improvement in quality of life. ^99m^Tc-LD showed higher levels of tumour uptake as compared with surrounding tissues.

Malignant pleural mesothelioma (MPM) is a primary tumour arising from the mesothelial cells of the pleura and is associated with aggressive local tumour invasion and poor prognosis (Connelly *et al*, 1987). This represents a serious health problem as the worldwide incidence continues to increase in the Western Europe and the United States (Antman, 1980). In the United States ∼2300 new cases are diagnosed annually, increasing up to 50% the prevalence rates in the last 10 years (Price, 1997). In the United Kingdom, is responsible for 2700–3000 deaths each year and a prevalence increase is estimated until 2020 (Peto *et al*, 1995). In Mexico, it is difficult to determine the real incidence of MPM due to disease under diagnosis and non-registration; this results in only 1500 registered cases in 15 years (Prevalencia del mesotelioma pleural en México, 2007). Eighty percent of patients with MPM have a history of asbestos exposure (Ashcroft, 1973; Nicholson *et al*, 1982) other risk factors include history of thoracic radiation (Hodgson *et al*, 2007) and possibly exposure to Simian virus 40 (Rizzo *et al*, 1999; Cristaudo *et al*, 2005). There are three main histological subtypes of MPM: epithelial, sarcomatoid, and mixed. The epithelial type is the most common and represents 50% of all cases, whereas sarcomatoid is the most aggressive type and represents 15% of all cases (Winslow and Taylor, 1960).

Surgery is the principal modality in the curative intent treatment of patients with MPM and would be administered with only a palliative goal or, especially in those with localised disease. The procedures included among these surgeries are: pleurectomy and decortication; and extrapleural pneumonectomy (EPP; Yan *et al*, 2009). Extrapleural pneumonectomy consists of cytoreductive surgery on pleural surfaces while preserving the lung, this procedure is recommended for patients without tumour involvement of lung parenchyma or soft tissues; morbidity is 10% and the mortality is <1% (Baldini *et al*, 1997). To date, there is no consensus concerning the role of the surgery in MPM (de Perrot *et al*, 2009). Moroever, some studies suggested that the radical treatment has a better prognostic (Treasure *et al*, 2011). As a consequence, several studies have explored the use of multimodality therapy, including adjuvant and neoadjuvant chemotherapy, even in patients with resectable disease (Weder *et al*, 2007).

For non-resectable advanced MPM, systemic chemotherapy has shown to improve disease control and to diminish associated symptoms. The use of single-chemotherapy agents has reported an overall response rate (RR) in the range of 10–20% (Kindler, 2008; Ray and Kindler, 2009). Meta-analysis has demonstrated that the most beneficial drug in advanced MPM treatment is cisplatin (Berghmans *et al*, 2002). Recent studies have reported that use of combined chemotherapy exhibited a higher RR compared with single-agent chemotherapies (Ray and Kindler, 2009). The combination of cisplatin and pemetrexed was established as first-line standard treatment after the results of a phase III trial that compared the combination *vs* cisplatin alone and reported higher median overall survival (OS) (Vogelzang *et al*, 2003).

Doxorubicin has demonstrated to be an active drug in the treatment of advanced MPM in phase II studies, as well as the combination with cisplatin, with RR of 25–46% and median OS of 8.8–10 months. Unfortunately, long-term use of doxorubicin is limited because of its toxicity profile such as grades 3 and 4 myelosuppression, mucositis, nausea, vomiting, alopecia, and cardiotoxicity (Henss *et al*, 1988; Ardizzoni *et al*, 1991; Chahinian *et al*, 1993).

Liposomal doxorubicin (LD), doxorubicin hydrochloride encapsulated in liposomes coated with methoxy polyethylene glycol, has shown diminished uptake by the reticule–endothelial system, a longer half-life, a different toxicity profile from that of non-polyethylene glycolylated liposomes, and theoretically increases liposomal deposition in tumour tissue. A phase II European Organisation for Research and Treatment of Cancer (EORTC) study that evaluated LD (45 mg m^−2^) as monotherapy, showed a median survival of 13 months and only mild toxicity; the EORTC members concluded that there are good reasons for evaluating this drug in combination with other cytostatic drugs (Baas *et al*, 2000).

We have conducted a trial to evaluate the safety, progression-free survival (PFS), OS, and RR of LD plus cisplatin in patients with advanced MPM, as well as ^99m^Tc-LD distribution in MPM lesions after chemotherapy administration.

## Patients and methods

### Patients

In this Phase II study, patients with histologically confirmed stage IIIB/IV MPM were included. All biopsies were centrally reviewed by an expert panel of oncology Pathologists. A set of immunohistochemical stains was used in all cases. Patients had the following Inlcusion criteria: Eastern Cooperative Oncology Group performance status of 0–2; no prior chemotherapy treatment; age ⩾18 years; normal haematological, renal and hepatic functions (white blood cell count ⩾1500 mm^−3^, haemoglobin ⩾10.0 g dl^−1^, platelet count ⩾100 000 mm^−3^, total bilirubin ⩽1.5 mg dl^−1^, aspartate aminotransferase ⩽2.0 mg dl^−1^ normal upper limit, creatinine ⩽1.5 mg dl^−1^); measurable disease according to the modified Response Evaluation Criteria in Solid Tumours (RECIST) gauge for assessment of response in MPM (Byrne and Nowak, 2004) and life expectancy >12 weeks. A complete medical history and physical examination including complete blood count with differential and platelet count, biochemical profile, urinalysis, electrocardiogram, and axial computed tomography (CT) of the chest and abdomen were obtained. The study protocol was approved by the local Institutional Scientific and Bioethics Committee (007/024/OMI-CV/304/06) and was registered in clinical trials (NCT00886028). All patients signed written informed consent.

### LD radiolabelling and scintigraphic imaging procedure

**Figure 1:**
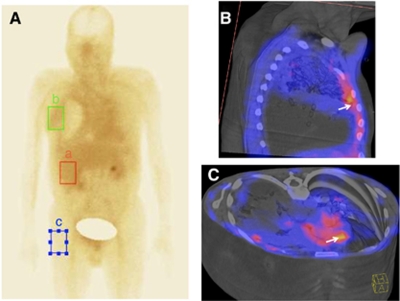
(**A**) Whole-body scan acquired 1 h after ^99m^Tc-LD injection. The image shows the ROIs as described in the text by the (a) Liver, (b) Tumoural tissue, and (c) Soft tissue (background area). Single-photon emission CT images in figures (**B**) and (**C**) show ^99m^Tc-LD uptake in tumour tissue (arrows).

The individual dose of LD (Doxopeg, Asofarma-México, México DF, Mexico) was radiolabeled with ^99m^Tc using SNS/S *N*,*N*-Bis(2-mercapto-ethyl)-*N*′,*N*′-diethyl-ethylenediamine (BMEDA) as described previously by Bao *et al* (2004). The labelling efficiency of the ^99m^Tc-BMEDA was calculated by utilising the activity in ^99m^Tc-LD before and after separation in the column. To evaluate biodistribution and accumulation of ^99m^Tc-LD in measurable tumour tissue, 1 h after the infusion, the patient underwent planar whole-body imaging (10 cm min^−1^, 1056 × 1056 matrix, no zooming) and a thorax (single-photon emission CT (SPECT) (64 frames 30 s^−1^; 64 × 64 matrix, no zooming) using a double-head SPECT camera (e-Cam, Siemens, Erlangen, Germany). Single-photon emission CT images were fused with low-dose CT images (Sensation 16, Siemens) for anatomical reference. Standard regions-of-interest (ROI) were drawn on the planar and SPECT images in tumour, soft tissue, and the liver to evaluate ^99m^Tc-LD accumulation. Figure 1 depicts ROI on whole-body images and the relationship between them. Uptake index was calculated as the ratio of the number of counts in ROIs in tumour and soft tissue *vs* liver.

### Chemotherapy administration

Patients received LD 40 mg m^−2^ in 60 min and cisplatin 60 mg m^−2^ in a 3-h infusion on day 1 of every 21 days cycle, patients were treated for a maximum of six cycles and all patients received antiemetic therapy with ondansetron, dexamethasone, and aprepitant. Radiolabeled LD was administered only in the first cycle to verify LD accumulation in tumour tissue.

### Toxicity evaluation

Physical examination and laboratory tests were performed before every cycle. The National Cancer Institute Common Toxicity Criteria was used to evaluate toxicity. Dose reductions or delayed chemotherapy was permitted when toxicity grades 3–4 did not resolve after 1 week.

### Response assessment and follow-up

Response assessment was determined every two cycles according to the modified RECIST criteria (Byrne and Nowak, 2004). Patients with chemotherapy response were evaluated by a Thoracic Surgeon (JFC-C) to determine whether they were candidates for surgical treatment after four cycles of chemotherapy; if surgery was not possible, chemotherapy continued up to six cycles until disease progression, high-grade toxicity, or withdrawal of informed consent. Patients without disease progression during chemotherapy treatment were followed-up with axial CT every 2 months to determine PFS.

### Quality of life

The 30-item EORTC Quality of Life (QoL) Questionnaire (EORTC QLQ-C30) version 3.0 (Spanish version) was used in this trial. EORTC QLQ v3 consists of five multi-item functional scales, three symptom scales, a global health status/QoL scale, and six single items. Transformation of scores was performed according to the instructions in the manual. Scores on all scales and single items could range from 0 to 100 points. Higher scores on functional and global health status QoL scales reflect better functioning. On symptom scales, higher scores mean more symptoms or problems. Quality of life questionnaires were filled out 1 day before the first chemotherapy cycle and after finishing four cycles of chemotherapy.

### Statistical analysis

Continuous variables were descriptive with means, medians and s.d.; variable categories were proportions and 95% confidence intervals (95% CIs). Inferential comparisons were conducted by means of the Student's *t-* or the Mann–Whitney *U*-test according to data distribution (normal and non-normal, respectively) determined by the Kolmogorov–Smirnov test. The *χ*^2^ or the Fisher exact test was used to evaluate significance among categorical variables. Comparisons between QoL were performed before and after the second cycle of chemotherapy and were analysed with the Wilcoxon-related samples test. When the scale showed differences >10%, these were considered as clinically significant. Statistical significance was determined as a *P*-value (*P*⩽0.05) with a two-tailed test. Progression-free survival and OS were determined from day of initiation of chemotherapy until progression and until death or until last day of follow-up, respectively, and were analysed by the Kaplan–Meier test, while comparisons among patients with or without response were analysed with the log-rank test. The SPSS version 15 (SPSS Inc., Chicago, IL, USA) software package was utilised for data analysis.

## Results

### Population of patients

This trial included patients from two thoracic oncology reference centres in Mexico City (Instituto Nacional de Cancerología (INCan) and Instituto Nacional de Enfermedades Respiratorias). From September 2006 to September 2009, 38 consecutive patients with stage III/IV MPM were included. All included patients had unresectable disease. The median age at diagnosis was 60.1±11.5 years. Males represented the majority (73.6%) of patients. Seventy four percent of patients had epithelioid histology. A total of 84.22% of patients were classified as high-risk according to the EORTC risk classification (Table 1).

### Response rate

Two patients were unable to be valuable after treatment because of one mortality and the other unable to make the study to asses response. Partial responses were observed in 38.9% (14/36; 95% CI, 22.97–54.82) of patients, stable disease in 41.6% (15/36; 95% CI, 25.5–57.7), and disease progression in 19.4% (7/36, 95% CI, 6.48–32.31). No complete response was observed. Patients who responded to chemotherapy were re-evaluated by the Thoracic Surgeons department. From all patients with a response to treatment six patients were eligible for surgery, but only three patients had complete cytoreductive surgery.

### Toxicity evaluation

Severe acute toxic effects are listed in Table 2. There were no treatment-related deaths and only one patient withdrew from treatment secondary to side effects (neutropaenia without recovery). Two patients had an increase of creatinine levels and required a change from cisplatin to carboplatin. The most commonly experienced side effects were nausea and vomiting in 36.9% and hand–foot syndrome in 6 patients (15.8%). Toxicity grade ⩾2 comprised leukopaenia (13.2%), neutropaenia (7.9%), nausea and vomiting (36.9%), and anaemia (21.1%), in addition to hand–foot syndrome (7.9%).

### Survival outcomes

**Figure 2:**
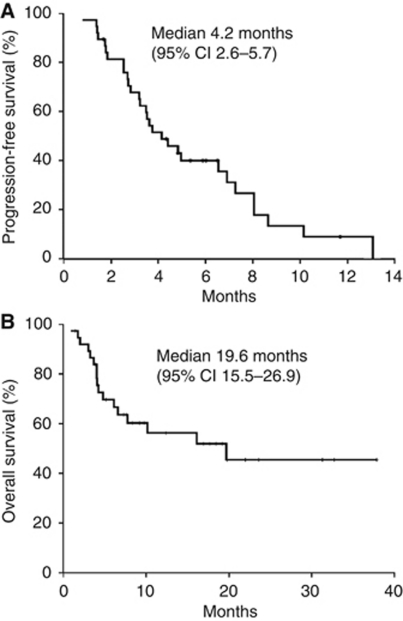
Kaplan–Meier of patients treated with chemotherapy and LD. (**A**) progression-free survival and (**B**) overall survival.

The median follow-up was 11.6±9 months. Median and mean number of received cycles of treatment were 4 and 3.5 cycles, respectively. Median time to disease progression was 4.2 months (Figure 2A; 95% CI, 3.4–5.9 months) and median OS was 19.6 months (95% CI, 15.2–37.2 months; Figure 1B). Patients who responded to the treatment had better survival than patients who did not. Patients who did not respond to chemotherapy had a median survival of 7.7 months (95% CI, 2.17–38.18 months) and those who responded did not reach OS at time of analysis (*P*=0.06). After progression to LD plus cisplatin, 35 (92.1%) and 27 (71.1%) patients received second-line chemotherapy based on pemetrexed, gemcitabine, or vinorelbine.

### Quality of life (QoL)

**Figure 3:**
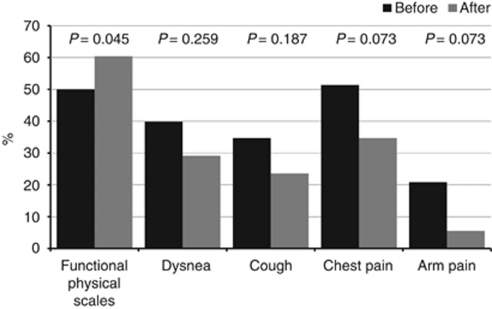
Percent of the functional physical scales, the dysnea, the cough, the chest pain and the arm pain before and after of the treatment.

QoL exhibited changes before and after two cycles of chemotherapy in functional physical scale, dysnea, cough, and chest–arm pain. Functional physical scale was the only one with statistical significant changes (*P*=0.045). However, dyspnoea, cough, chest pain, and arm pain decrease >10%, being statistically significant (Figure 3). Emotional, cognitive, social, fatigue, insomnia, hyporexia, constipation, diarrhoea, financial difficulties, haemoptysis, mucositis, dysphagia, and alopecia did not demonstrate statistical differences.

### Tissue distribution of ^99m^Tc-LD

The ^99m^Tc-LD labelling efficiency was 48±12%. Scintigraphical images (Figures 1B and C) depicted an uptake index of radiolabeled LD in the tumour tissue of 0.78±0.16%. In 80% of the imaged patients, tumour uptake index of ^99m^Tc-LD at 1 h after its administration was higher than uptake index in soft tissue (0.29±0.09% *P*<0.001).

## Discussion

Advanced MPM is a poor-prognosis tumour with a median OS of 4–13 months in patients without treatment and of 6–18 months in patients with palliative chemotherapy. In a meta-analysis conducted in 2002, cisplatin showed to be the most important chemotherapy drug for treatment of advanced MPM (Berghmans *et al*, 2002). After this study, the combination of cisplatin and new generation drugs has been investigated to determine its beneficial role in these patients. Only pemetrexed and raltitrexed in combination with cisplatin have been studied in phase III trials, showing better OS compared with cisplatin alone (Vogelzang *et al*, 2003; van Meerbeeck *et al*, 2005; Kelly *et al*, 2011). The combination of cisplatin and pemetrexed was established as first-line standard treatment after the results of a phase III trial that compared the combination *vs* cisplatin alone and reported higher median OS (12.1 *vs* 9.3 months; *P*=0.020), longer median time to disease progression (5.7 *vs* 3.9 months; *P*=0.001), and higher RR (41.3% *vs* 16.7% *P*=0.0001). In our institution the majority of patients, do not have access to social security or medical insurance. Because of pemetrexed costs is difficult for patients to have it as a standard care. Combinations of cisplatin with other effective drugs (vinorelbine, gemcitabine) have not, to our knowledge, been studied in phase III trials (Sorensen *et al*, 2008).

Doxorubicin have shown efficacy against mesothelioma (Antman, 1980). The concentration of LD in tumours is result of the enhanced permeability and retention effect, as a consequence of a leaky microvasculature and impaired lymphatics supporting the tumour area Drummond *et al*, 1999). The movement of LD into the mesothelioma interstitium its probably by extravasation through the discontinuous endothelium of the mesothelioma microvasculature. Once in the tumour, LD is mainly localised in the interstitum surrounding tumour cells (Figure 1). In the present phase II study, we evaluated the antitumour activity of a combination with LD plus cisplatin (LD/P) in patients with advanced MPM. Our results showed that this combination (LD/P) is active, with partial response in 38.9% and stable disease in 41.6% of patients, although the majority of patients have poor-prognosis characteristics (84.22%) according to the EORTC risk classification. In addition, toxicity was manageable, only one patient withdrew from treatment due to side effects, and there were no treatment-related deaths. Quality of life showed changes before and after the second cycle of chemotherapy in functional physical scale, dysnea, cough, and chest–arm pain. Liposomal doxorubicin is much less toxic (Uziely *et al*, 1995), and response had been reported in mesothelioma in 7% of patients as monotherapy with median time to tumour progression and survival of 5 and 12 months (Hillerdal *et al*, 2008a, 2008b), respectively. Antitumour activity of LD as monotherapy was noted in a small phase II study in which 15 patients with advanced MPM were treated with LD 55 mg m^−2^ every 4 weeks; 4 of the 15 evaluable patients showed objective response (26%) and a median OS of 13 months; QoL remained good during the study and no significant toxicity was observed, suggesting positive activity of this drug in MPM (Skubitz, 2002). Other phase II study published by the EORTC that included 33 patients with MPM evaluated LD as monotherapy with 6% of RRs, and similar to the previous trial, median survival was 13 months (Baas *et al*, 2000). The Nordic Mesothelioma Group investigated a combination of LD, gemcitabine, and carboplatin in MPM in a phase II trial, reporting 32.4% of RRs with median time to progression and OS of 8.6 and 13 months respectively, however, for patients with epithelioid subtype, median OS was 17 months (Hillerdal *et al*, 2008a, 2008b). Similar to our results, this group reported a correlation between response and survival.

Delivery and penetration of the chemotherapy drugs into tumours are limited by a number of factors related with altered stromal composition in neoplastic tissue. Mesotheliomas might contain large amounts of fibrous tissue, which might reduce drug penetration. We also evaluated ^99m^Tc-LD accumulation in MPM lesions after chemotherapy administration. As reported previously by several authors, in our study LD has shown a diminished uptake by the reticulo–endothelial system, a longer half-life, and a different toxicity profile than doxorubicin, the results have shown an increased liposomal deposition in tumour tissue, resulting in antitumoural effectiveness (Northfelt *et al*, 1996). It has been established that a mechanism of resistance of solid tumours to chemotherapy may comprise limited penetration of anticancer drugs into tumour tissue (Tannock *et al*, 2002). In our study, we found a similar distribution of radiolabeled LD in tumour tissue as compared with liver (uptake index 0.78±0.16%); the latter is the primary liposome uptake site. However, LD uptake was significantly higher in tumour cells as compared with soft tissue. This result supports the antitumour activity of this treatment regimen (LD/P). A study in Kaposi's sarcoma patients treated with LD also shows a correlation between distribution and chemotherapy response as compared with doxorubicin (Castagneto *et al*, 2005).

In conclusion, the combination LD plus cisplatin showed to be an active combination for MPM treatment with acceptable toxicity profile.

Because this is a phase II study with a small number of patients, it is necessary to perform phase III studies that compare the LD/P against cisplatin/pemetrexed or cisplatin/raltitrexed to determine a standard of treatment in first line.

## Figures and Tables

**Table 1:** Clinical characteristics of patients

**Characteristics**		**%**
Patients enrolled	38	100
		
*Gender*		
Male	29	76.6
Woman	9	23.7
		
*Age (years)*		
Median±s.d.	60.1±11.5	
		
*Smoking status*		
Non-smokers	16	42.1
Smokers	22	57.9
		
Asbestos	23	60.5
Wood smoke	12	31.6
		
*Stage at enrollment*		
IIIB	27	72
IV	11	28
		
*Histology*		
Epithelial	28	73.3
Sarcomatoid	6	15.4
Biphasic	4	10.3
		
*ECOG-PS*		
0	4	10.3
1	24	61.5
2	10	25.6
		
Albumin g dl^−1^	2.8±0.5	
Median received treatment	4	
Delayed treatment	16	42.1
		
*Response*		
Partial response	14	38.9
Stable disease	15	41.6
Progression	7	19.4
		
*EORTC*		
Good-prognosis group	6	15.78
Poor-prognosis group	32	84.22

Abbreviations: ECOG-PS=Eastern Cooperative Oncology Group-performance status; EORTC=European Organisation of Research for Cancer; Karnofsky=Karnofsky performance status.

**Table 2:** Toxic effects

**Toxic effect**	**Grades ⩾2 no. of patients**	**%**
Anemia	22	57.9
Nausea and vomiting (non-haematological)	14	36.9
Lymphopaenia	8	21.1
Thrombocytopaenia	8	21.1
Fatigue (non-haematological)	8	21.1
Leukopaenia	5	13.2
Anorexia (non-haematological)	5	13.2
Neuropathy (non-haematological)	5	13.2
Neutropaenia	3	7.9
Hand-foot syndrome	3	7.9
Creatinine	2	5.3
Constipation (non-haematological)	2	5.3
Diarrhoea (non-haematological)	1	2.6
